# Hennebert’s sign due to intralabyrinthine schwannoma – case report

**DOI:** 10.1007/s00405-026-10238-4

**Published:** 2026-05-05

**Authors:** Raymond van de Berg, Milou van Orsouw, Walter Jakub Szweryn, Jérôme Joseph Waterval, Alida Annechien Postma, Henricus Petrus Maria Kunst, Joost Johannes Antonius Stultiens

**Affiliations:** 1 Department of Otorhinolaryngology and Head and Neck Surgery, School for Mental Health and Neuroscience, Faculty of Health Medicine and Life Sciences, Maastricht University Medical Center, Maastricht, The Netherlands; 2 Dutch Academic Alliance Skull Base Pathology, Maastricht University Medical Center & Radboud University Medical Center, Maastricht/Nijmegen, The Netherlands; 3 Department of Radiology and Nuclear Medicine, School for Mental Health and Neuroscience, Faculty of Health Medicine and Life Sciences, Maastricht University Medical Center, Maastricht, The Netherlands; 4 Department of Otorhinolaryngology and Head and Neck Surgery, Radboud University Medical Center, Nijmegen, The Netherlands

**Keywords:** Intralabyrinthine schwannoma, Hennebert sign, Fistula test, Nystagmus, Cochlea, Vestibule, Vestibular schwannoma, Pressure induced nystagmus, Valsalva, Inner ear schwannoma

## Abstract

**Objective:**

To illustrate how careful examination can reveal signs that may be elicited by an intralabyrinthine schwannoma.

**Patients:**

A patient referred to a tertiary dizziness referral clinic.

**Intervention(s):**

The patient underwent extensive vestibular assessment, audiometry, and imaging, including computed tomography (CT) and contrast-enhanced magnetic resonance imaging (MRI).

**Main outcome measure(s):**

Signs of central or vestibular pathology were assessed, including the fistula test, as well as audiometric thresholds and imaging abnormalities.

**Results:**

The patient exhibited unilateral vestibular hypofunction and profound hearing loss in the left ear. Application of a Politzer balloon to the left external auditory canal induced a combination of upbeat nystagmus and horizontal nystagmus to the left, particularly upon pressure release. The CT scan showed no abnormalities. MRI revealed a schwannoma located in the basal and middle turns of the left cochlea extending into the vestibule and in close contact with the stapes.

**Conclusions:**

This case report provides unique evidence of Hennebert’s sign in a patient with an intralabyrinthine schwannoma, highlighting the importance of performing detailed vestibular testing and considering this diagnosis.

**Supplementary Information:**

The online version contains supplementary material available at 10.1007/s00405-026-10238-4.

## Introduction

Hennebert’s sign, or pressure-induced nystagmus, can be found when pressure on the external auditory canal (e.g. tragal compression, Politzer balloon) or increased middle ear pressure (e.g. Valsalva maneuver) results in nystagmus. This nystagmus can be observed in several disorders of the inner ear, which allow transmission of the externally applied pressure into the inner ear. The resulting inner ear pressure changes lead to stimulation or inhibition of the vestibular organ, depending on the endolymphatic flow which is created [1]. Hennebert’s sign can occur in various inner ear disorders. This case report demonstrated Hennebert’s sign in a patient with an intralabyrinthine schwannoma, underscoring a potentially underrecognized clinical sign.

## Case presentation

A 54-year old woman was referred to our tertiary clinic for vestibular disorders. Her relevant medical history included single-sided deafness in her left ear since 15 years and a period of episodic vertigo attacks 13 years ago. An MRI of the cerebellopontine angle, made in another hospital at that time, was without abnormalities and no conclusive diagnosis was made. She was recently analyzed again because of new-onset vertigo. These symptoms included two main different types of vertigo: 1) vertigo when exerting pressure on her left auditory canal with her finger, and; 2) short-lived attacks of vertigo, triggered by e.g. turning around in bed and looking up, without any accompanying symptoms. A recent CT scan of her temporal bone was without abnormalities. No conclusive diagnosis was made and she was referred to our tertiary clinic.

**Fig. 1:**
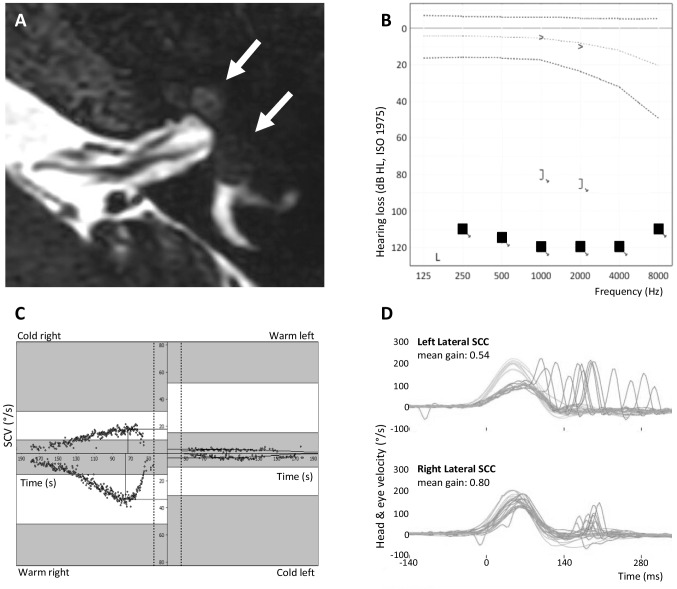
**a**-**d** Test results of the presented patient with an intralabyrinthine schwannoma. a: MRI T2-weighted axial image demonstrating the intralabyrinthine schwannoma in the left inner ear: white arrows indicate the absence of high-intensity signal in the cochlea (top arrow) and vestibule (bottom arrow). **b** Audiogram demonstrating profound hearing loss in the left ear (black squares with arrow indicate that the tone was not heard). **c** Caloric test demonstrating low-frequency vestibular hypofunction of the left horizontal semicircular canal. **d** Video Head Impulse Test demonstrating vestibular high-frequency hypofunction of the left horizontal semicircular canal. dB HL: decibel hearing level; SCV: slow component velocity; SCC: semicircular canal

**Fig. 2:**
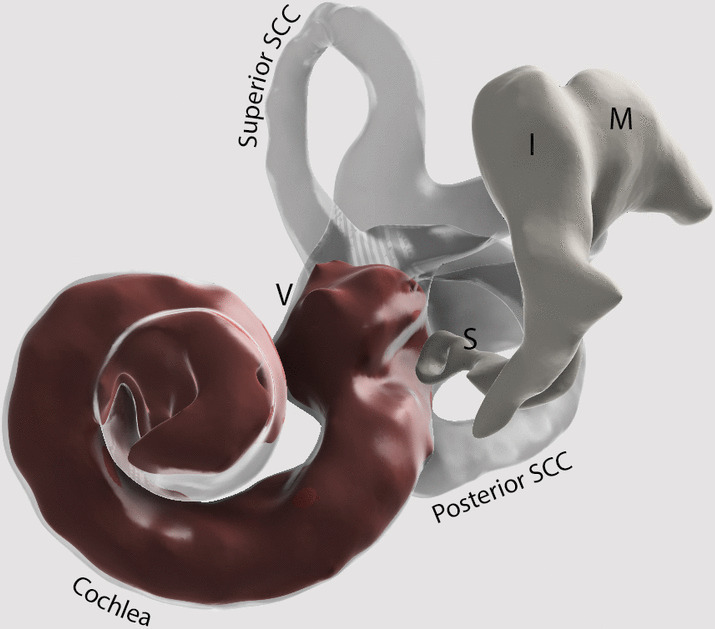
The intralabyrinthine schwannoma (in red) in the left inner ear, seen in close relation to the ossicular chain. SCC: semicircular canal; V: vestibule; S: stapes; I: incus; M: malleus. Image created from fused CT and T2-weighted MRI images, using 3D Slicer (open source) and Fusion 360 (Autodesk, San Francisco, CA, USA) [11]

History taking confirmed the different types of vertigo, although the second type of dizziness was not present on the day of her visit to our clinic. Relevant findings of physical examination included anisocoria, negative Dix-Hallpike maneuvers and supine rolls, a positive head impulse test to the left and Hennebert’s sign. The latter, measured with Video Frenzel Goggles (VisualEyes, Interacoustics, Middelfart, Denmark), involved a predominantly horizontal drift of the eyes to the left and right with some small nystagmus beats to the left, when the patient exerted and released pressure on her left external auditory canal with her finger (Supplementary Video 1). When using the Politzer balloon in her left ear, a mix of upbeat nystagmus and horizontal nystagmus to the left was found, especially when pressure was released (Supplementary Video 2). Audiometry revealed normal hearing in her right ear (modified Fletcher index 1–4 kHz of 13 dB HL) and profound hearing loss in her left ear (Fig. 1b). The caloric test showed a vestibular hypofunction on her left side, with an asymmetry of 78% (Fig. 1c). This was congruent with Video Head Impulse Test findings: vestibulo-ocular reflex gains of 0.80 (right) and 0.54 (left) (Fig. 1d). A CT scan of the temporal bone was reperformed in high resolution (0.4 mm slice thickness) and demonstrated no abnormalities. Additionally, a contrast-enhanced MRI scan of the cerebellopontine angle (including hydrops protocol) was ordered. Objective was to investigate a possible inner ear disorder related to the stapes footplate, which could lead to vestibular stimulation by applying pressure on the external auditory canal (e.g. hydrops) [2]. The MRI scan showed an intralabyrinthine schwannoma in the vestibule and in the basal and middle turn of the cochlea on the left side (intravestibular-cochlear type [3]) (Fig. 1a). The semicircular canals were not involved. The schwannoma was in close contact with the stapes footplate (Fig. 2). Retrospectively, the schwannoma could also be observed in the non-contrast-enhanced T2-weighted MRI which was performed 13 years before. In these 13 years, the schwannoma increased in size from initially occupying the middle and basal turn, to extension into the vestibule.

The Hennebert’s sign, deafness, and vestibular hypofunction were diagnosed as being related to the intralabyrinthine schwannoma in the left inner ear. Management involved a wait and scan approach, because of the slow tumor growth and the limited impact of the pressure-induced dizziness on her daily life. The short-lived dizziness with head movements was diagnosed as an episode of Benign Paroxysmal Positional Vertigo in remission, which did not reoccur during follow-up. The MRI scan did not show any additional abnormalities. The patient was referred to the neurologist for further analysis of the mild anisocoria. No signs of lesions along the sympathetic trunk or other significant abnormalities were found.

## Discussion

This case report provides clear evidence of Hennebert’s sign and a likely mechanism in a patient with an intralabyrinthine schwannoma. This implies that in patients with symptoms and signs suggesting a perilymphatic fistula, without abnormalities on a high-resolution CT scan, a contrast-enhanced high-resolution MRI of the inner ear should be considered.

An intralabyrinthine schwannoma is a benign tumor which results from the Schwann cells in the inner ear [4]. It is probably an underreported cause of sensorineural hearing loss [5]. Diagnosis can be challenging, since the symptoms and results of physical examination and audiovestibular testing can be various and non-specific. Symptoms may include (fluctuating and/or progressive) sensorineural hearing loss, tinnitus, aural fullness, vertigo, motion-induced dizziness and unsteadiness [3–6]. In most patients, physical examination is without abnormalities. Audiometry often reveals a sensorineural hearing loss and vestibular testing can demonstrate decreased or absent vestibular responses [4, 6]. The case reported here is unique, since it clearly demonstrated a manifestation of this intralabyrinthine schwannoma during physical examination: Hennebert’s sign.

Hennebert’s sign, or pressure-induced nystagmus, is generally believed to mainly result from a third window mechanism of the inner ear. Etiologies of these third windows are for example perilymphatic fistula, superior canal dehiscence syndrome and dehiscences between the otic capsule and surrounding structures. However, other etiologies, not related to a third window, were also previously described, like Menière’s disease, vestibular atelectasis, a hypermobile stapes footplate or stapes surgery [1, 7, 8]. In Menière’s disease and vestibular atelectasis, it is hypothesized that the stapes footplate is brought in contact with the membranous labyrinth, respectively due to distension (hydrops) or collaps of the membranous labyrinth [1, 9]. In the case presented here, the close relation of the stapes footplate to the intralabyrinthine schwannoma most likely plays a significant role in the Hennebert’s sign. After all, it can be hypothesized that by applying pressure on the external auditory canal, the stapes footplate is able to mobilize the intralabyrinthine schwannoma. As a result, this tumor could create an endolymphatic flow resulting in vestibular stimulation, or it could directly stimulate the sensory structures by pressing with its volume against these structures. Additionally, the tumor might have (partially) mobilized the stapes footplate, resulting in a perilymphatic fistula [7]. Regarding the latter, it should be noted that intralabyrinthine schwannomas spreading to the middle ear or displacing the stapes footplate, seem to be relatively rare [4].

In case of Hennebert’s sign, a high-resolution CT scan of the temporal bone is the preferred first imaging modality, to detect the presence of a bony defect in the otic capsule. Nevertheless, only < 60% of the affected sites seem to be detected in patients with a surgically identified labyrinthine fistula [1]. Using a gray-scale invert function, it might be possible to visualize the stapes footplate and any potential defects [7]. However, this case illustrates that if the CT scan is negative, it may be valuable to perform an MRI scan. Additionally, in this case, the presence of ipsilateral sensorineural hearing loss warranted MRI imaging. This imaging should include thin-section high-resolution T2-weighted images, preferably combined with post-gadolinium T1-weighted images [5]. On MRI T2-weighted images, an intralabyrinthine schwannoma replaces the high signal intensity which is normally found in the labyrinth. After administration of gadolinium, an enhancement of the tumor is observed on MRI T1-weighted images. These T2 and post-gadolinium T1-weighted images correlate well in case of an intralabyrinthine schwannoma. In the case presented here, this diagnosis was unfortunately missed 13 years earlier. This emphasizes that radiologists should also be aware of the existence of an intralabyrinthine schwannoma and specifically examine the inner ear for such an abnormality, instead of only ruling out pathology in the cerebellopontine angle [5]. If the T2 and post-gadolinium T1-weighted images would show no abnormality, it could be considered to add an MRI-hydrops protocol [10], to investigate the presence of vestibular hydrops or vestibular atelectasis as a possible origin of Hennebert’s sign. In the presented case, the CT-scan and all MRI scans were ordered at the same time for logistic reasons.

Treatment of an intralabyrinthine schwannoma depends on the symptoms, size, location and growth of the tumor. Management options include a wait-and-scan approach, surgical removal, or radiotherapy. A conservative wait and scan strategy may currently be preferred in most cases, even in tumors demonstrating slow growth [4, 6]. Surgical removal is indicated in a minority of patients, mainly because of disabling vestibular symptoms, tumor size and/or significant tumor growth [4, 6]. Radiotherapy seems to have no significant effect on vertigo [6]. In cases of therapy-resistant vertigo attacks, chemical labyrinthectomy (e.g. intratympanic gentamicin injection) could be considered to ablate vestibular function [3]. This may be particularly favorable when serviceable hearing is present. In the current case, the patient opted for a wait-and-scan management, as the pressure-induced dizziness had a limited impact on her daily life and she preferred to avoid ablation of residual vestibular function. In patients with single-sided deafness due to an inner ear schwannoma, cochlear implantation (with or without tumor removal) may be considered [12, 13], depending on local feasibility. This option was not pursued in the present case, because cochlear implantation for single-sided deafness is currently not reimbursed in the Netherlands.

## Conclusion

Hennebert’s sign can be present in patients with an intralabyrinthine process, such as a schwannoma in this case. Contact between the stapes footplate and an intralabyrinthine schwannoma might explain pressure-induced nystagmus and/or vertigo. Therefore, a high-resolution T2-weighted MRI of the inner ear, complemented with contrast-enhanced high-resolution T1-weighted images should be considered in patients with symptoms and signs possibly indicating a perilymphatic fistula, but without any abnormalities on a high-resolution CT scan of the temporal bone.

## Supplementary Information

Below is the link to the electronic supplementary material.

Supplementary Video 1 (MP4 4061 KB)

Supplementary Video 2 (MP4 5275 KB)
